# Multilevel Factors for Life Satisfaction Among Residents in Non-Urban Subsidized Senior Housing

**DOI:** 10.1093/geroni/igaa057.360

**Published:** 2020-12-16

**Authors:** BoRin Kim, Sojung Park, Casey Golomsky, Marguerite Corvini, Allison Wilder, John Wilcox, Allysha Winburn

**Affiliations:** 1 University of New Hampshire, Durham, New Hampshire, United States; 2 Washington University in St. Louis, St. Louis, Missouri, United States; 3 University of New Hampshire, Durham, United States; 4 University of West Florida, Pensacola, Florida, United States

## Abstract

Although a quarter of HUD-assisted properties for older adults are located in rural/non-metropolitan areas, there is limited understanding of the population living in these locations. Advanced age and low income are known risk factors for poor physical and mental health. Older adults in rural subsidized housings may be at increased risk for poor health and social isolation due to their isolated locations and small-scale housing complexes. This presents the additional challenge of service provision for the residents’ needs. This study aims to explore multi-level factors affecting life satisfaction among residents in subsidized senior housing. Data were collected for five subsidized senior housings in New Hampshire: two in Coos county (rural, population=33,055) and three in Strafford county (suburban, population=128,613). Mixed-methods approaches were used: Community/organizational-level data were collected using semi-structured interviews conducted with the directors of senior housings. At the individual level, quantitative survey data were collected from 82 residents of five senior housings. Contrary to expectations, we found that residents in rural senior housings were likely to report better life satisfaction (Coef.=0.597, p<.01) than those in suburban areas despite controlling for individual-level factors such as age, gender, education, marital status, health, social relations, and service use. The most salient terms used in the interviews with directors of rural senior housings include limited resources, tight community, and emotional support. The last two may be protective factors positively influencing life satisfaction among their residents. Our results contribute to development strategies to improve quality of life among residents in rural/non-metropolitan subsidized senior housing.

